# Building a programme theory of a specialist paediatric palliative and hospice care programme: development process and methodological reflection

**DOI:** 10.1186/s12904-024-01492-6

**Published:** 2024-07-20

**Authors:** Martin Wallner, Daniela Haselmayer, Martin Nagl-Cupal, Jasmin Eppel-Meichlinger, Hanna Mayer

**Affiliations:** 1 Division of Nursing Science with focus on Person-Centred Care Research, Karl Landsteiner University of Health Sciences, Dr.-Karl-Dorrek-Strasse 30, Krems an der Donau, 3500 Austria; 2 Faculty of Social Sciences, Department of Nursing Science, University of Vienna, Alser Strasse 23/12, Vienna, 1080 Austria

**Keywords:** Complex interventions, Programme theory, Theory-driven evaluation, Paediatric palliative care, Programme evaluation (MeSH), Hospice and palliative care nursing (MeSH), Palliative care (MeSH), Paediatrics (MeSH)

## Abstract

**Background:**

Paediatric palliative and hospice care aims to improve the quality of life of children with life-limiting and life-threatening conditions and their families. The number of these patients has risen significantly in recent years, resulting in an increased need for palliative care for this population. Although the need for paediatric palliative and hospice care is growing, meaningful outcome evaluation to demonstrate its effectiveness as a complex healthcare intervention is in its early stages. For complex interventions (programmes), theory-based evaluations have grown in prominence in recent years. They seek to understand how and why an intervention works by uncovering its underlying mechanisms by means of programme theory. To support both outcome evaluation in paediatric palliative care and a reflective practice of programme theorizing, we aimed to describe the construction of a programme theory for a specialist paediatric palliative and hospice care programme in Austria and to offer a reflective account of its development process.

**Methods:**

We drew on a combination of theory-based evaluation frameworks to construct a programme theory consisting of an action and a change component. Through multiple iterations, incorporating different stakeholders’ perspectives and drawing on different sources of knowledge and theory, we theorized how and why the programme likely achieves its intended outcomes.

**Results:**

The programme theory outlines the proposed chains of events, causal mechanisms and outcomes of a specialist paediatric palliative and hospice care programme for children and families in several areas corresponding to its main conceptual tenets. Through a range of activities and interventions, the programme triggers coping and adaptation mechanisms that ultimately contribute to family and child wellbeing in physical, psychological, social, and spiritual dimensions. Established trust and partnership between children/families and healthcare professionals as well as a person-centered and family-centered approach were identified as enabling factors.

**Conclusions:**

Our findings provide insights into how a specialized paediatric palliative and hospice care programme works to achieve its intended outcomes for children and families. This helps demonstrate its impact, contributing to meaningful outcome evaluation and service improvement.

## Background

Paediatric palliative and hospice care aims to improve the quality of life of children with life-limiting and life-threatening conditions and their families by preventing and relieving suffering, whether physical, psychological, social or spiritual [1]. Meeting the needs of this vulnerable population is considered a growing global health concern, as the number of children with life-limiting and life-threatening conditions and associated palliative care needs is rising internationally. It is therefore considered important to understand how palliative care provides benefits to this population [2]. Providing paediatric palliative and hospice care requires a comprehensive approach, with the input of a skilled multidisciplinary team [3]. These healthcare professionals are embedded in social contexts, including hospital and community services, that are influenced by local and national policies. While the need for paediatric palliative and hospice care is growing, meaningful outcome evaluation to demonstrate its effectiveness as a complex healthcare intervention is in its early stages [2].

In the past years, there has been extensive discussion on the development and evaluation of complex healthcare interventions [4–8]. An intervention is considered complex if it consists of multiple interacting components, targets a range of behaviour changes, involves several target groups and settings, and requires expertise and skills of the people delivering the intervention [5]. Other criteria include flexibility pertaining to intervention delivery and adherence, as well as interaction between the intervention and the context [9]. Interventions of this kind are also referred to as programmes or social programmes. A social programme is defined according to Rossi, Lipsey and Freeman [10] as “[…] organized, planned, and usually ongoing effort designed to ameliorate a social problem or improve social conditions”.

Theorizing a programme is considered key in its development phase [4]. This is recognised with increasing emphasis in nursing and healthcare research [9, 11]. A multitude of methodological guidance has been published on developing and evaluating complex healthcare interventions [4, 8, 9], including theory-based evaluations [6, 7, 11, 12], with programme theory as its central element [13]. In its most recent iteration, one influential framework, that of the UK Medical Research Council [5], included an increased focus on programme theory, supporting, among others, a theory-based evaluation perspective [9]. In it, Skivington, Matthews, Simpson, Craig, Baird, Blazeby, Boyd, Craig, French, McIntosh, et al. [9] emphasised not only the need for prospective but also for retrospective development of programme theory. After a programme has been developed and implemented, retrospective development of a programme theory is essential to uncover the implicit theoretical basis, and to elaborate how the intervention could be evaluated.

Theory-driven evaluations seek not only to understand if a programme works, but also how it works to mitigate a social problem. This is to be achieved by theorizing and modelling the path from an intervention to the intended change [13, 14]. Programme theory is an explicit theory or model of how an intervention, such as a project or programme, contributes to a chain of intermediate results and finally to the intended outcomes [14].

Moreover, programme theories draw on different sources of knowledge from different perspectives, including, importantly, contextualized, practical knowledge of practitioners [14], also referred to as stakeholder theory [13] in theory-based evaluation.

Programme theory development can be a lengthy, iterative process that involves abductive and/or retroductive reasoning to devise a theory that can explain observed facts [15]. Exactly how the theorizing process is to be achieved, however, is subject of debate [16], urging scholars to call for a reflective practice of programme theorizing to strengthen its conceptual and technical foundations [17, 18].

The research described in this article set out to construct a programme theory of an existing specialist paediatric palliative and hospice care programme (SPPHC) in one federal state of Austria, Europe, which went into operation in 2014.

To pursue this aim we investigated how and why a specialist paediatric palliative and hospice care programme leads to its intended effects by means of programme theory. In this paper, we describe the construction of the programme theory and offer a reflective account of the process to contribute to unravelling the “magic box” [16] of programme theorizing.

## Methods

With this research we drew on the methodology of theory-driven evaluation [19] as well as realistic evaluation [20]. Programme theory is the most important constituent feature of theory-based evaluations [13, 16] and commonly consists of two components: a theory of action (action model) and a theory of change (change model). A theory of action outlines which interventions need to be undertaken by who and for which target group, whereas a theory of change articulates the effects and events triggered through the implementation of interventions resulting in the intended change [14]. Realistic evaluation pays particular attention to context as enabling factor for programme mechanisms to be triggered effectively. Consequently, this programme theory aimed to include both an action and a change component, with a special focus on context.

### The programme and its setting

The focus of the programme theory development was on the specialized paediatric palliative and hospice care programme. The programme, which went into operation as such in 2014, was composed of individual services that had already been established independently of each other at that time. It was implemented based on a concept proposed by a group of experts [21]. This concept draws on recommendations from the IMPaCCT consensus statement of paediatric palliative care which outlines core standards to meet the needs of dying children [3], and which have recently been updated to GO-PPaCS [22]. The programme is embedded within the wider context of a general paediatric palliative and hospice care programme in Lower Austria and includes inpatient as well as homecare hospice and palliative care services in addition to basic care and respite services. Lower Austria is one out of nine federal states in the Central European republic of Austria (total population of Austria is approximately 9.05 million), with a population of approximately 1.71 million people in 2022 [23].

### Process of programme theory construction

The process was informed by a combination of conceptual frameworks of theory-based evaluation [19, 20] and drew on different sources and types of theory. This included stakeholder theory as well as scientific theory [13], while also building on existing programme theory [2] and concepts [18]. We informed the process of programme theory construction at all phases by continuously identifying and reviewing relevant scientific literature. In doing so, we aimed at providing a general overview at the beginning, and definitions of used terms, assessing the impact of palliative care. Main goals of the literature review were supplying a basis for the interviews and deepening the results.

**Fig. 1:**
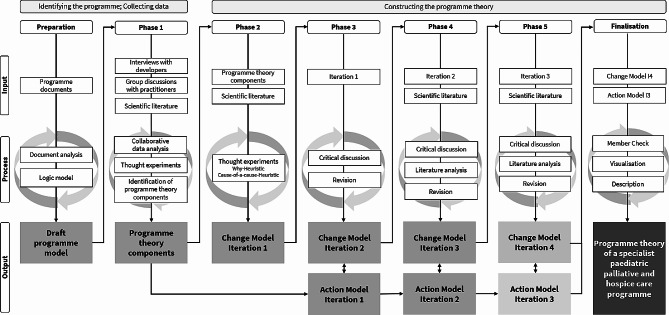
Process of programme theory construction

The process of programme theory construction (Fig. 1) lasted from March 2020 to May 2021 and involved three main steps: (a) identifying the programme (preparation; phase 1), (b) collecting data (preparation; phase 1), and (c) constructing the programme theory (phases 2–5; finalisation). The identification of the programme resulted in a draft programme model and the identification of programme theory components. Through several iterations, we developed a change model (phase 2–5) and an action model (phase 3–5).

#### Identifying the programme

Identifying the programme’s interventions and exploring options for its evaluation is an important first step when building a programme theory for existing interventions [9]. Therefore, we drew on various sources of knowledge to gain insights into the programme. On a conceptual level, we aimed at identifying the mission and aim of the programme, carefully reviewing its inherent structure to be compatible with conceptual definitions of a social programme or complex intervention.

#### Collecting data

To inform the construction of the programme theory, we synthesized knowledge from different perspectives and different sources, to understand what interventions have what effects, and what mechanisms explain the effects and the ultimate outcome. We gathered data using the following methods: (1) programme document review, (2) individual interviews with programme developers (*n* = 9), and (3) group discussions (*n* = 3) with paediatric palliative and hospice care practitioners (*n* = 16).

1. Programme document review

For preparation, we retrieved and reviewed the available programme documents to identify the programme and gain an overview of its different components. We screened these documents for programme components and for underlying assumptions regarding mechanisms of action. On this basis, we identified and visualised the rough programme structure using a logic model template [24]. This resulted in a draft model of the programme. In this early stage of the theory construction process the assumptions on why the interventions were effective were vague and related to structural, process and outcome criteria of the IMPaCCT statement concerning “good palliative care” [3]. We identified preliminary components in three broad conceptual areas in the programme documents: (1) psychosocial aspects, (2) physical symptoms, and (3) dying and grieving. However, we found gaps in knowledge regarding the exact interventions and activities of the programme. In addition, there was no information on mediating factors that influence the impact of the programme activities. We used this draft model to guide interviews with programme developers and group discussions with practitioners from the different services of the programme and to conduct a focussed search in the scientific literature.

2. Individual interviews with programme developers

Interviews with programme developers are an important method for explicating the implicit assumptions of a programme [14, 25]. We therefore aimed at filling knowledge gaps regarding the development of the programme, uncovering implicit assumptions about the mechanisms of the programme and identifying concrete measures to achieve the defined goals. Using a semi-structured interview guideline, we individually interviewed people (*n* = 9) with different roles in the development process of the programme. We conducted nine virtual interviews from May to June of 2020. The guide contained various topics, including the development history of the programme, planned interventions and objectives, coordination and cooperation, funding, staff and their qualifications. The guideline was used flexibly for each interview partner, with a focus on their respective expertise. Each interview was conducted by one person of the project team, while a second person took written notes. In a first step, we read the transcribed interviews to get closer to the content. In the second step, we re-read them and organised and coded them using categories based on the logic model, pertaining to pre-conditions, plan, implementation, and outcomes of the programme [24]. These findings formed the basis for a subsequent focused literature review and informed the planning of the group discussions with practitioners.

3. Group discussions with specialised paediatric palliative and hospice care practitioners

Group discussions with practitioners aimed to uncover programme-related interventions, outcomes, and possible mechanisms of impact. For this purpose, multi-disciplinary teams from the Paediatric Hospice Team, the out-patient Paediatric Palliative Care Team, and the in-patient Paediatric Palliative Care Unit participated in the group discussions (*n* = 3) which were held in-person in July and August of 2020. The participants (*n* = 16) were registered nurses and physicians with advanced training in paediatric palliative care, physicians with advanced training in palliative care and managers of the respective teams.

We then processed and analysed the data collaboratively in the research team. Using thought experiments, we identified programme theory components from the data. Finally, we organised and categorised the data using different logic models templates [24, 26].

#### Constructing the programme theory

Programme theory construction took place through an iterative process, as suggested in the procedural model [16]. This model emphasises the concept of abduction as a ‘missing link’ between deduction and induction in programme theory construction and proposes to facilitate abductive reasoning using methods such as thought experiments. To build programme theory, we formed a team of researchers with methodological and theoretical expertise both in theory-based evaluation, family nursing, and paediatric care to build the programme theory.

#### Change model

We constructed the first iteration of the programme theory by building the change model incrementally starting from the endpoint. The change model of the programme theory outlines central chains of events, causal mechanisms, and outcomes of the programme in several areas corresponding to the main conceptual tenets of palliative care. Thereby we drew on the identified components of the programme (phase 1). The principle of prioritizing outcomes (‘do the outcomes first’) [14, 26] and the so-called *why heuristic* as well as the *cause of a cause heuristic* [27] guided us in phase 2. We derived the endpoint (wellbeing) conceptually and adopted it from the definition of palliative care by the World Health Organization [1]. To identify programme mechanisms, we focused on mediating factors. Mediators explain why the ultimate outcome is achieved. They are present along the entire chain of events, i.e., from the beginning of the programme [16] and explain the relationship between concepts or variables and the ultimate outcome.

In phase 3, we discussed iteration 1 of the change model critically in the research team by systematically examining it in terms of logic, plausibility, and conceptual adequacy. Identified conceptual ambiguities and logical weaknesses of this iteration were subsequently revised.

#### Action model

Starting from phase 3 and onwards, the first iteration of the action model was constructed, drawing on the framework by Chen [19] and using the programme theory components identified in phase 1. The action model includes six main components: (1) implementing organisations, (2) target population, (3) programme implementers, (4) intervention protocol, (5) partner organisations and (6) environmental context (external programme factors) [19]. In phase 4 and 5 we critically discussed the subsequent iterations and corresponding revisions of the programme theory (change model and action model).

#### Finalising the programme theory

In the final phase, we subjected the action model and change model to a member check [28] in May of 2021. The programme participants had the opportunity to discuss the models in terms of coherence, plausibility [14] and adequacy. The feedback from the programme participants (*n* = 11) did not result in a need to revise the models. We then prepared the final models as figures and transformed the components and interrelationships into a complete programme theory consisting of narrative description and visual representation. The result is an iteratively matured programme theory, which can be used to plan the evaluation of specialised paediatric palliative care in Lower Austria.

### Ethical considerations

We followed the ethical principles of research and assured adherence using an informed consent form: keeping all data anonymous and confidential, explaining that participation was voluntarily and the possibility to withdraw participation at any time without negative consequences or giving reasons. Participants agreed to being audio recorded and its use for further analysis. We encouraged participants to contact the project team with any questions.

Approval from an ethical board was not obtained, as according to Austrian law non-interventional studies do not require approval, we did not involve vulnerable groups and individuals, and did not identify any risks for physical or psychological harm to participants.

## Results

**Fig. 2:**
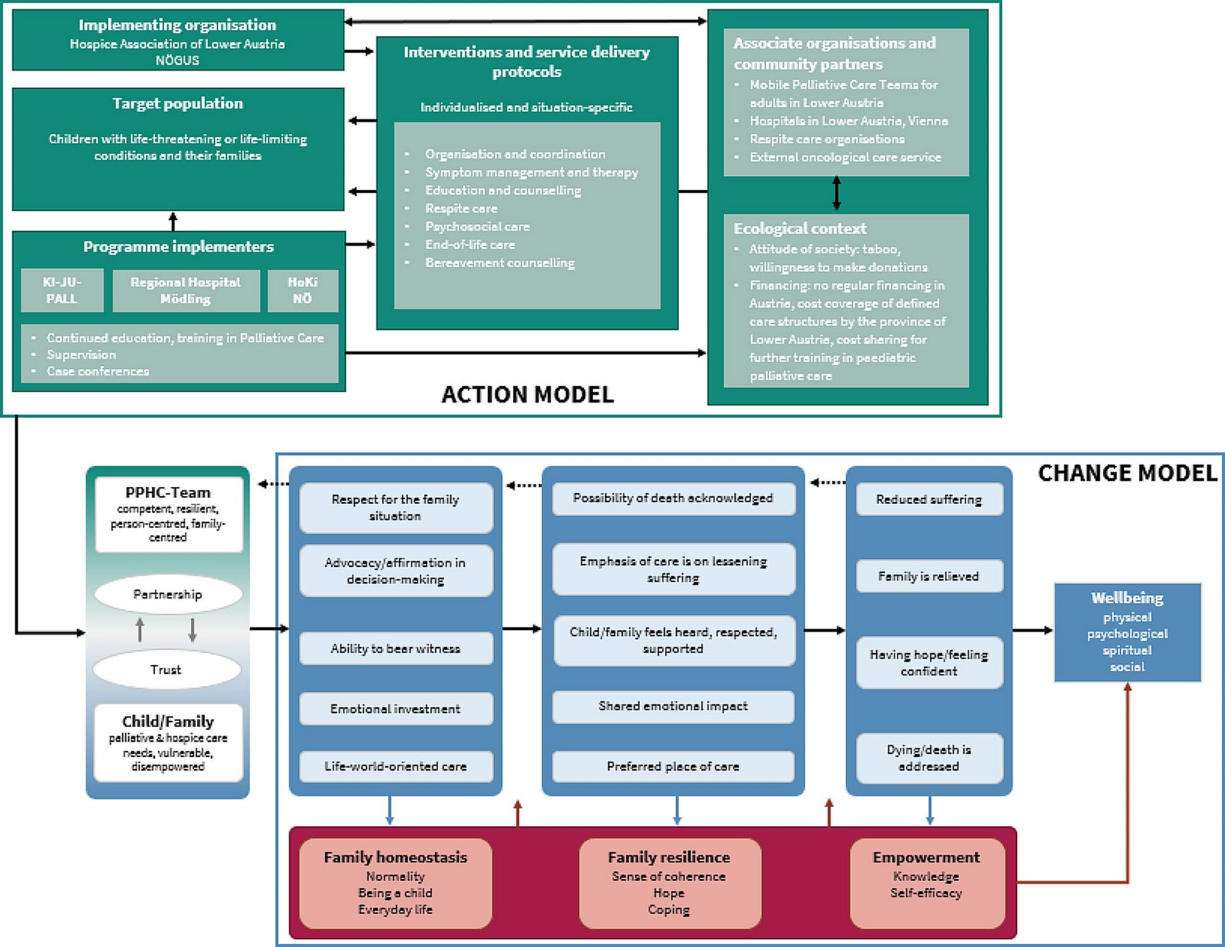
Logic model of the programme theory

We developed a programme theory of a specialist paediatric palliative and hospice care programme, consisting of an action model and a change model. A logic model of the programme theory is depicted in Fig. 2.

### Action model

The action model illustrates how the programme operates to achieve the desired outcomes. It explains how processes are set in motion to bring about the expected changes in the target group, as outlined in the change model.

#### Implementing organisations

The structures and processes of the programme have grown over time, resulting in the development of a separate, practically feasible programme for SPPC in Lower Austria. The interventions of the programme are distributed among two homecare services (*Paediatric Hospice Team* and *Paediatric Palliative Care Team*) and one in-patient service (*Paediatric Palliative Care ward in a hospital*).

#### Target population

Specialized Paediatric Palliative Care (SPPC) was created for the care of children, adolescents, and young adults with life-limiting and life-threatening illnesses (groups according to the IMPaCCT statement).

#### Programme implementers

The people involved in the implementation of the programme are attributed as being innovative, motivated and family-oriented. These attributes were necessary to build up the aforementioned care structures, establish them sustainably and thus close the gap in care for the target group. The interventions are carried out by a multi-professional team (e.g., nurses, physicians, psychologists, social workers, therapists) as well as by volunteers. Important characteristics of these persons are competence, resilience, person-centredness, family-centredness, qualified by appropriate further advanced training in paediatric palliative care and having the opportunity to participate in case discussions and supervision. The persons work together in a multi-professional and cross-organisational way and try to ensure continuity of care for the families.

#### Intervention protocol

It is a characteristic of the interventions that they are tailored according to the individual needs and situations of children and their families. Very few interventions can be described in quantitative terms of frequencies, specific intervals, or duration. This is partly due to the wide spectrum of children’s illnesses in terms of symptoms and course of illness. Another reason lies in the different living situation of the child (e.g., size of the housing unit) socioeconomic aspects (e.g., education, income) as well as culture, norms and habits of the families (e.g., dealing with illness, distribution of roles). In addition, dependence on infrastructure is an issue as well (e.g., distance to the hospital).

The interventions of the programme can be summarised with the following categories: (a) organisation and coordination, (b) symptom management, (c) nursing tasks, (d) education and counselling, (e) respite care, (f) psychosocial support, (g) end-of-life care and (h) bereavement care. Each of these categories encompass numerous individual activities that are carried out by the professionals and volunteers on an individual basis and depending on the situation.

#### Partner organisations

Cooperation with partner and peer organizations are equally important. These include educational and health care providers in the local district, temporally well accessible hospitals and organizations with respite services for the whole family.

#### Environmental context (external programme factors)

With regard to the environmental context, it can be noted that the issues of limited life expectancy and dying of children and adolescents are a taboo subject. There is also nonregular funding for hospice and palliative care. Programme costs are covered by the local government and the services are free of charge for families in need.

### Change model

Through a range of activities and interventions, which are outlined in the action model, the programme triggers coping and adaptation mechanisms, which ultimately contribute to family and child wellbeing in physical, psychological, social, and spiritual dimensions. The change model consists of four main components: (1) Pre-condition (context), (2) Chains of events, (3) Mediators, and (4) the ultimate outcome (Fig. 2).

The change model is read from left to right, which is indicated in the model with solid black arrows. Dashed arrows from right to left indicate feedback or interaction dynamics. Arrows in blue (from top to bottom) and arrows in red (from bottom to top) indicate reciprocal dynamics between chains of events and the underlying mechanisms or mediators.

#### Pre-condition

This component of the change model shows which conditions must be fulfilled and which prerequisites must be present on the part of the staff for the change to be set in motion. This part of the change model draws on the notion of realistic evaluation [20] that certain conditions need to be in place for the mechanisms to be triggered effectively.

Our empirical data from group discussions with programme practitioners revealed that trust and partnership between palliative care practitioners and the child with the life-threatening or life-limiting condition and its family must be established as a pre-condition. Parents who are in a vulnerable, disempowered situation together with their child trust in the professional expertise of the programme team. This trust is possible if the staff is competent (qualified/trained for paediatric palliative and hospice care) and has both a family-centred and a person-centred attitude. This was also supported by recent literature [2]. We furthermore identified a holistic, family-centred, and lifeworld-oriented approach as important pre-conditions of effective paediatric palliative and hospice care. The staff is resilient and therefore able to endure the situation together with the parents and to participate emotionally. Staff respect the parents’ situation and encourage or represent them in decision-making. This strengthens trust and in the long term creates a relationship.

#### Chains of events

The chains of events show how (dynamics) and through what (content) the ultimate outcome is achieved. They outline possible sequences of events and intermediary outcomes of the target group. They can be read in various combinations and arranged in different order, both within and between the identified areas. This is illustrated by the feedback loops (dashed arrows from right to left).

The first segment articulates how the practitioners’ preconditions are expressed in mutual and collaborative action with the family/young person. This is reflected in the SPPC team’s respect for the family situation. Care and support are life-world oriented. The SPPC team acts as an advocate and encourages the family in decision-making. In this process, the SPPC team members show emotional commitment and involvement and are able to endure the situation because they are professionally competent and resilient.

Next are situations and events experienced by families as a result of mutual action with the programme staff. The starting point for the programme is a life-threatening or life-limiting illness of a child. In this case, the parents are confronted with the fragility of the child’s life and its finiteness, which occurs at different speeds depending on the clinical condition.

Parents grieve for their child’s health, feel responsible for their child’s wellbeing and (re-)adapt their hopes and expectations for parenthood and the future.

The family experiences a sense of vulnerability, develops coping strategies and thus adapts to the situation. Over time, an understanding of the possibility of the child’s death emerges and the family, which over time develops expertise in the child’s illness and the impact on the family’s situation, is able to focus care on reducing suffering (rather than curing) [2].

Parents of a child with a life-threatening illness see themselves as their child’s protectors and find themselves caught in a conflict: they neither want their child to suffer, nor do they want their child to die. They find it difficult to accept that the child will die, which would be necessary to focus their care on reducing suffering. In this situation, advocacy, and support in decision-making from practitioners are essential. Staff need to be able to be emotionally participative and endure the situation together with the parents in order to adequately care for them in this situation [2]. In this way, the family feels heard, respected, and supported and the difficult emotional experience can be shared with the help of professional support (‘shared suffering’).

Through this family-centred, lifeworld orientation of care and support, the family can be or remain in the preferred/situationally appropriate place.

These in turn lead to the next segment, i.e. outcomes corresponding to basic principles of the programme: suffering is reduced, the family is relieved, hope/confidence is developed, and dying/death is addressed. In sum, these sequences of events lead to the intended outcome/endpoint of the programme (wellbeing).

#### Mediators

Our empirical data suggested that the paediatric palliative and hospice care programme triggers adaptation and coping mechanisms, which we identified as *family homeostasis*, *family resilience*, and *empowerment*. These mechanisms explain *why* the programme leads to the ultimate outcome of wellbeing.

##### Family homeostasis

Empirical literature emphasises that a family has the capacity or tendency to regain stability (status quo). Family homeostasis describes this process. However, this is individually defined, and each family has different antecedents that disturb the balance and also different homeostatic mechanisms/abilities/tendencies to regain this equilibrium/stability. These processes are described in the literature in a setting-specific way depending on the clinical picture, family status etc. [29]. Changes in one part of the family system must be followed by compensatory changes in other parts. In daily life, there are numerous changes ranging from temporary irritations to long-term conflict or stress in the family life cycle. The family will try to restore its previous state of equilibrium; however, if this is not possible, a new state of equilibrium will be adopted through homeostatic mechanisms. This new state of equilibrium can come about through medium and long-term outcomes. Homeostasis is thus a driving force or resistance in the face of change. As each family develops its own equilibrium and homeostatic mechanisms, different families may present their conflicts or dysfunctions differently, even when dealing with the same level of dysfunction [29].

In the process of developing programme theory, we identified three essential aspects pertaining to family homeostasis: *normality*, *everyday life* and *being a child*.

##### Family resilience

For this research, resilience can be described as a family functioning trait, i.e. an ability to maintain a balance between change and stability within the family [Patterson, 1995, cited in 30]. Thus, family resilience involves more than dealing with stressful conditions, carrying a burden or overcoming a challenge. This approach recognises the potential for personal and relational transformation and growth that can be developed out of adversity. By developing key resilience processes, families can emerge stronger and more resourceful through collaborative efforts [31]. Peer and Hillman [32] studied stress and resilience in parents of children with cognitive and physical disabilities. They showed that social support, optimism and coping are relevant resilience factors.

In the development of our programme theory, we also identified aspects that can be associated with the concepts of *sense of coherence*, *coping* and *hope*.

##### Empowerment

Empowerment is an approach of encouraging people to discover their own strengths and to achieve a higher degree of autonomy and self-determination [33]. It is described as a concept, a process or an outcome. As a concept, it can be applied at different levels: Macro level (policy) or micro levels (groups/individuals). The empowerment process is described as a social process, a helping process, and a dynamic process. Participation, shared decision-making, trust, openness, and acceptance in nurturing environment are some fundamentals for empowerment. Empowerment includes self-development and self-efficacy, better self-understanding, hope for the future, personal growth, inner satisfaction, and connectedness [34].

A sense of control is an outcome of empowerment. Frustration and distress are negative consequences of empowerment. Establishing trusting, ‘nurturing’ and respectful relationships between healthcare-teams, caregivers and affected persons, providing support, encouragement, and information. The descriptive themes of empowerment address intrapersonal aspects (e.g., loss of control, powerlessness, need for competence) and interpersonal aspects (e.g., partnership, interaction, trust, support). Trust and participatory relationship are described in several studies as a consequence of the empowerment process. Many other publications refer to the need for a trusting, open, accepting and respectful relationship as a precondition for starting an empowerment process [35]. Parents need people in the palliative and hospice care team who understand, are mindful of and know coping strategies for their worries, fear of loss and the parent-child relationship [36, 37].

#### Ultimate outcome

Together, chains of events and mediators outline and explain how and why the palliative and hospice care programme leads to the endpoint of wellbeing and thus how and why it is effective. The World Health Organization defines palliative care as the prevention and alleviation of suffering for adult and paediatric patients and their families facing the problems of a life-threatening or life-limiting illness. These problems include the physical, psychological, social and spiritual suffering of patients and the psychological, social and spiritual suffering of family members [38]. The (re)establishment of wellbeing in the aforementioned areas is thus the goal of palliative care, and accordingly, wellbeing was conceived as the endpoint of the programme theory.

## Discussion

The purpose of this article was to report and reflect on the development process of a programme theory of a specialist paediatric palliative care programme. Our programme theory proposes a framework articulating how and why a paediatric palliative and hospice care programme in Lower Austria leads to wellbeing for children and their families. We developed it systematically through several iterations, incorporating different stakeholder’s perspectives, and drawing on different sources of knowledge and theory. The programme theory comprises of two components, including both descriptive and prescriptive elements: the action model articulates the interaction of the persons involved, carrying out different activities and interventions and thus trigger the change that is outlined in the change model, which results in the desired outcome. It outlines how and why the programme leads to the intended outcome, thereby including both configurational and mechanism-based explanations [18]. Conceptually, the programme theory links fundamental principles of palliative care with established and elaborated concepts from family nursing literature as explaining mechanisms.

Our programme theory builds on the conceptual framework of programme theory as proposed by Chen [19], and also draws on ideas from realistic evaluation [20]. Moreover, our programme theory builds on existing programme theory for paediatric palliative care by Mitchell, Bennett, Morris, Slowther, Coad and Dale [2], which resulted from a realist review. Our programme theory includes an action model in the understanding of programme theory according to Chen [19], which is specific to the concrete setting and the care situation in Lower Austria. Drawing not just on existing programme theory, but also on established concepts from the family nursing literature, expert knowledge from programme developers as well as contextualized and experiential knowledge from practitioners, allowed us to propose a way of explaining how specialized paediatric palliative and hospice care functions in this case. Rather than just accumulating or referencing concepts, by theorizing the programme we articulated the complex patterns and interrelations between these phenomena and showed, for instance, how nurses, with their holistic and person-centred focus, skilfully operate and activate health and illness response patterns in persons with care needs and their social and environmental contexts. This relation-sensing quality of nursing was outlined by Bender [39] as being central to its unique effectiveness.

Methodologically, the proposed programme theory integrates two different conceptual frameworks of theory-based evaluation [19, 20], utilizing the strengths of both to fit the evaluand and the specific setting. While the conceptual framework of programme theory as outlined by Chen [19] provided the overall rationale and structure of this programme theory, ideas from realistic evaluation [20] particularly came to bear with regarding the focus on context as enabling factor for the programme mechanisms to be triggered effectively. Although each of these frameworks use different terms and concepts, theory-based evaluations share a common philosophical foundation in a realist theory of science [40].

We termed contextual considerations as pre-conditions in this programme theory. While the importance of context for social programmes is widely recognized, different conceptual frameworks articulate different notions of context using different terms, such as “pre-condition”, or simply “factors” [18]. The “pre-condition” component of this programme theory addresses one kind of context particular to paediatric palliative and hospice care, as was also proposed by Mitchell, Bennett, Morris, Slowther, Coad and Dale [2]. Chen [19] also addresses contextual factors in the action model component of his conceptual framework of programme theory, which are termed “ecological context”. In an attempt to avoid confusion, we adopted the term pre-condition to refer to contextual factors associated with the change component of programme theory, drawing on the realist notion of context [20], while leaving the “ecological context” in the action model labelled as is.

Until recently, programme theory construction, from a methodological point of view, had remained somewhat mysterious, with some using the term “magic box” to describe this unknown process [16]. This is also in line with our observation that the development of programme theories is rarely reported and explicitly described, as for example by Dössegger, Weibel, Frei, Wissmath and Hense [25]. A procedural model for programme theory construction proposed by von Werthern [16] is one recent contribution to the methodological discourse on programme theory construction. The procedural model draws attention to the epistemic principle of abductive reasoning and emphasises its important role in the process of programme theory construction to identify explaining mechanisms of a programme. The relevance of abduction for the analytically supported and methodologically controlled process of programme theory construction has already been highlighted by Dössegger, Weibel, Frei, Wissmath and Hense [25] and Lemire, Whynot and Montague [18].

To the best of our knowledge, outside from its initial utilisation by its creator, this is the first time the model was used to inform and report on the development process of a programme theory. We translated von Werthern’s [16] prescriptive procedural model into a model showing concrete processes of programme theory construction. This was to highlight and make explicit actual procedures, methods, and processes of theory construction, including heuristic devices used, such as logic models, analytical procedures, such as thought experiments (e.g., why heuristic), as well as discursive and communicative processes, such as critical discussion and revision of the programme theory. Mediators, i.e., outcome explaining factors, also referred to as programme mechanisms, were identified through ‘spontaneous’ and facilitated abductive reasoning during the process of data analysis and programme theory construction. According to the procedural model, abductive reasoning can be facilitated using thought experiments [16], such as the ‘why heuristic’ and the ‘cause of a cause heuristic’ [27]. While it is hard to delineate exactly how or when abductive reasoning occurred both during the process and retrospectively, having this concept in mind did serve as welcome frame of mind helping to demystify the ‘magic box’ of programme theory construction [16], particularly compared to less formalized ways of theorizing. On the other hand, given the demanding task of programme theorizing, researchers may find themselves overwhelmed at the face of having to consider additional formal aspects, which might inhibit creativity in the process of theorizing. So while this can be considered a welcome addition to enhance the conceptual and technical foundations of programme theorizing, as called for by Whynot, Montague and Lemire [17], it is not entirely without trade-off [16].

As reported in previous programme theory research in nursing [41], the action model presented no difficulties to build. This is likely due to the fact that we retrospectively identified the programme theory of an existing programme with established structures, processes, and routines.

Constructing the change model, on the other hand, presented major challenges. Arguably the most difficult step was getting started with constructing the change model from the collected data and finding the right fit or form, which took several attempts. Existing logic model templates, such as that of the W. K. Kellogg Foundation [26], outlining a linear programme logic from input to outcome/impact, proved to be of limited utility in this regard and for this particular programme. While this may be a usual ‘trial and error’ process to go through, it might help articulating such early difficulties to alleviate pressure, such as when the logic of a programme turns out not to be as self-evident or self-explanatory as initially assumed.

The process of programme theory construction was concluded after a certain number of iterations. Aside from timely considerations predicated by the study protocol, this was justified formally by having reviewed all available data and programme theory sources, and conceptually, in that the research team agreed on having achieved a preliminary satisfying degree of plausibility as well as explanatory and heuristic value. It was considered unlikely that producing more iterations would add value unless new data was collected.

A welcome side effect of the development process was that practitioners saw their work reflected in the programme theory. This became evident during a member check, indicating the potential of programme theory of being impactful beyond the scope of evaluation and research. Previous research also pointed toward the additional benefits of participatory programme theory development, including raised awareness for the positive changes practitioners bring about, a positive image of their work, mutual understanding between researchers and practitioners [41], and a sense of ownership of the programme [14]. This is of particular importance to the nursing discipline, which sometimes struggles to articulate and demonstrate its unique contribution to patient wellbeing [39], let alone provide causal explanations.

The programme theory was developed specifically for the present programme and its context in one region of Austria. Findings are thus limited in terms of external validity. Nevertheless, we assume that our programme theory with its various components can inform other programme theories in some regard. While highly contextualized aspects, due to their low level of abstraction, may be less suitable for this, the identified mediators possibly lend themselves to being transferred to other contexts, and may serve as a starting point for building programme archetypes [14, 18]. However, this requires further research and methodological reflection.

As with all theories, this programme theory will have to demonstrate how well it can explain observable facts pertaining the programme it set out to describe. This is to be investigated in the course of an evaluation study. The programme theory can be used to conceive an evaluation design and select appropriate outcome measures and methods, and to interpret evaluation findings in a meaningful way. Evaluation findings will inform both service improvement as well as the revision of the programme theory, adopting a long-term perspective of knowledge accumulation [18].

## Conclusions

Our findings provide insights into how a specialised paediatric palliative and hospice care programme works to achieve its intended outcomes for children and families. This helps demonstrating its impact, contributing to meaningful outcome evaluation and service improvement.

Programme theorizing and theory-based evaluations offer one way of strengthening the theoretical basis of nursing by demonstrating how it contributes to positive health trajectories for patients. Constructing programme theory is a demanding task that requires fundamental skills in theory and model building [16, 19], which should not be underestimated. Our reflective account articulates concrete processes of theory construction and may help to avoid pitfalls. Judging by the learnings and insights that are to be gained in the process, we argue that it is a rewarding undertaking.

## Data Availability

The datasets generated and/or analysed during the current study are not publicly available due to a commitment to protect study participant privacy but are available from the corresponding author on reasonable request.
